# Endoscopic transcavernous approach for functional pituitary adenomas

**DOI:** 10.1007/s00701-024-06168-x

**Published:** 2024-06-19

**Authors:** Daniele Starnoni, Roy T. Daniel, Mahmoud Messerer

**Affiliations:** 1 Department of Clinical Neurosciences, Neurosurgery Service and Gamma Knife Center, Centre Hospitalier Universitaire Vaudois (CHUV), Lausanne, Switzerland; 2 Faculty of Biology and Medicine (FBM), University of Lausanne (UNIL), Lausanne, Switzerland; 3 Department of Neurosurgery, University Hospital of Lausanne, Rue du Bugnon 46, Lausanne, CH-1011 Switzerland

**Keywords:** Cavernous sinus, Endoscopic approach, PitNet, Pituitary adenomas

## Abstract

**Background:**

Invasion of the CS is one of the limiting factors for total resection for PitNet tumors with cure rates less than 30%. Extended approaches may be considered in selective and well-studied cases of secreting adenomas.

**Method:**

We describe the key steps of the endoscopic transcavernous approach for functional pituitary adenomas with a video illustration. The surgical anatomy is described along with the advantages and limitations of this approach.

**Conclusion:**

A detailed knowledge of CS anatomy and familiarity with this surgical approach acquired in the laboratory is essential. Proper instrumentation is critical to decrease the risks of vascular injury.

**Supplementary Information:**

The online version contains supplementary material available at 10.1007/s00701-024-06168-x.

## Relevant surgical anatomy

The parasellar section of the internal carotid artery (ICA) consists of the cavernous and clinoid segments. The cavernous segment is a continuation of the paraclival segment at the level of the short vertical segment where the artery has an oblique trajectory posteriorly and delimits the inferior compartment of the cavernous sinus (CS), which is bordered medially by the medial wall of the CS, posteriorly by the short vertical segment, and superiorly by the horizontal segment of the ICA. The anterior genu of the ICA and the horizontal segment delimit the superior compartment of the CS, superiorly and laterally this compartment is delimited by the roof of the CS, the clinoid and oculomotor triangle. The posterior compartment is bounded by the short vertical segment, the posterior genu and the posterior wall of the CS. The inferior pituitary artery is located in this compartment, it has an infero-medial trajectory in the direction of the dorsum sellae. It may arise independently from the short vertical segment or as a branch of the meningohypophyseal trunk. The abducens nerve lies in the lowest portion of this compartment and runs parallel, just inferior, to the horizontal segment of the ICA at the level of the lateral compartment [5]. Several ligaments insert at the level of the medial wall of the CS [10]. The strongest and most consistent is the carotidoclinoid ligament (CCL), which is part of the proximal dural ring and fuses with the interclinoid ligament. Other ligaments are the inferior (IPL) (connecting the medial wall with the anterior wall of the SC), superior (SPL) (connects the medial wall with the horizontal portion of the ICA) and posterior (PPL) (in close anatomical relationship with the inferior pituitary artery) parasellar ligaments.

## Description of the technique

**Fig. 1:**
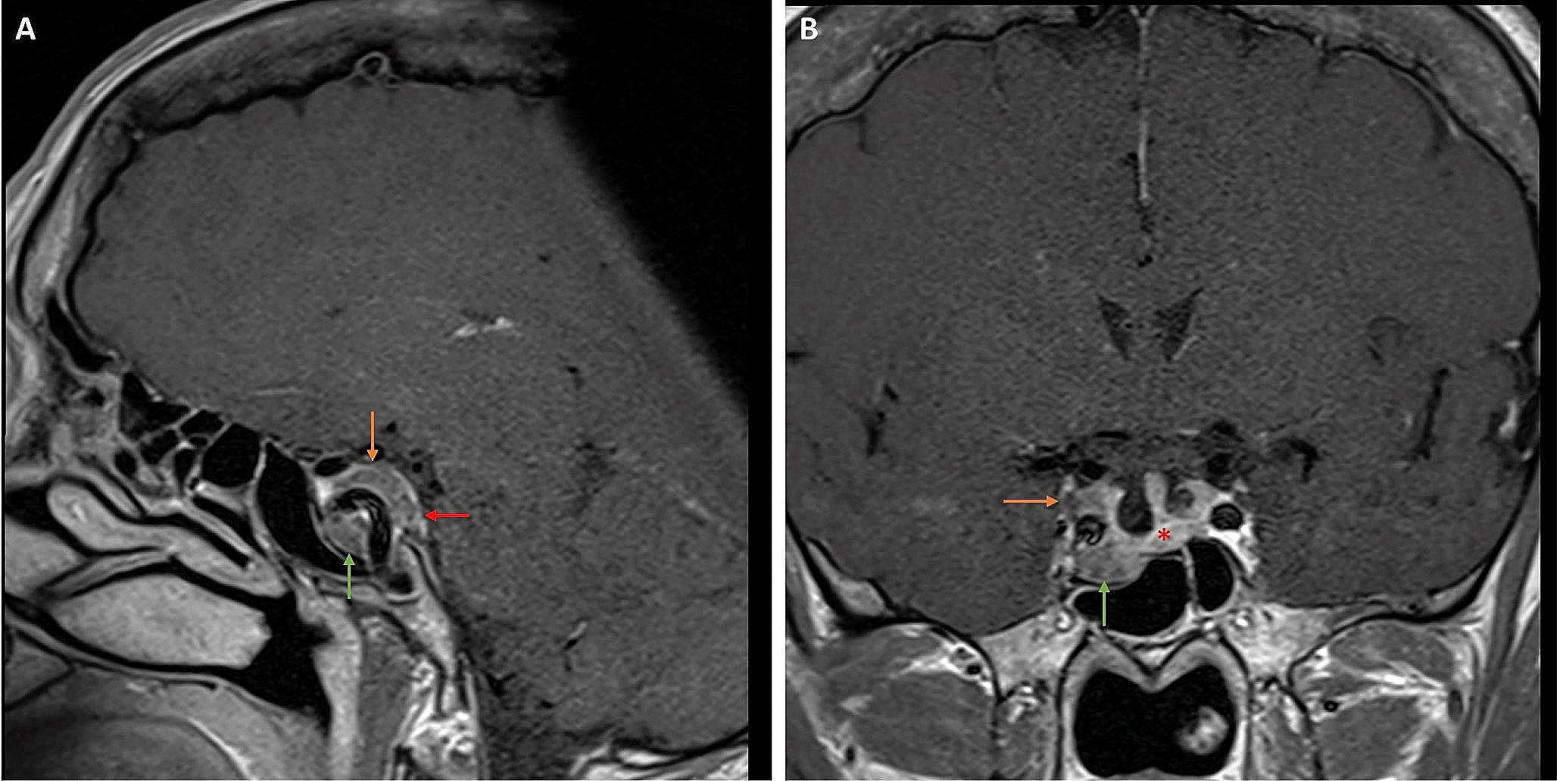
Case involving a 62-year-old patient with abnormal IGF levels (745 µg/L) and acromegalic features. (A and B) Pre-operative Gd-enhanced T1W sagittal and coronal MR images showing a pituitary macroadenoma invading the inferior (green arrow), medial, superior (orange arrow) and posterior (red arrow) compartments of the right cavernous sinus. Pituitary gland (red star) is slightly displaced to the left side

The patient is placed in a supine position with the head resting on a horseshoe head-holder and turned approximately 15 degrees toward the right shoulder and 10–15 degrees of neck flexion. Topical mucosal decongestants are used. Magnetic surgical navigation based on a preoperative computed tomography (CT) scan and a magnetic resonance imaging (MRI) is used(Fig. 1). For tumors projecting predominantly to one side of the sella and extending into the CS, the contralateral uninostril approach is used [1], given that exposure across the midline to the contralateral sella and CS area is consistently wider than to the ipsilateral side [6]. The middle turbinate is gently displaced laterally with a Cottle elevator in order to expose the junction of the keel of the sphenoid and the posterior nasal septum. The posterior septal mucosa is cauterized and incised with a bipolar cautery and reflected laterally to expose the ipsilateral sphenoid ostium. The posterior nasal septum is then displaced off the midline to allow exposure of the contralateral ostium.

**Fig. 2:**
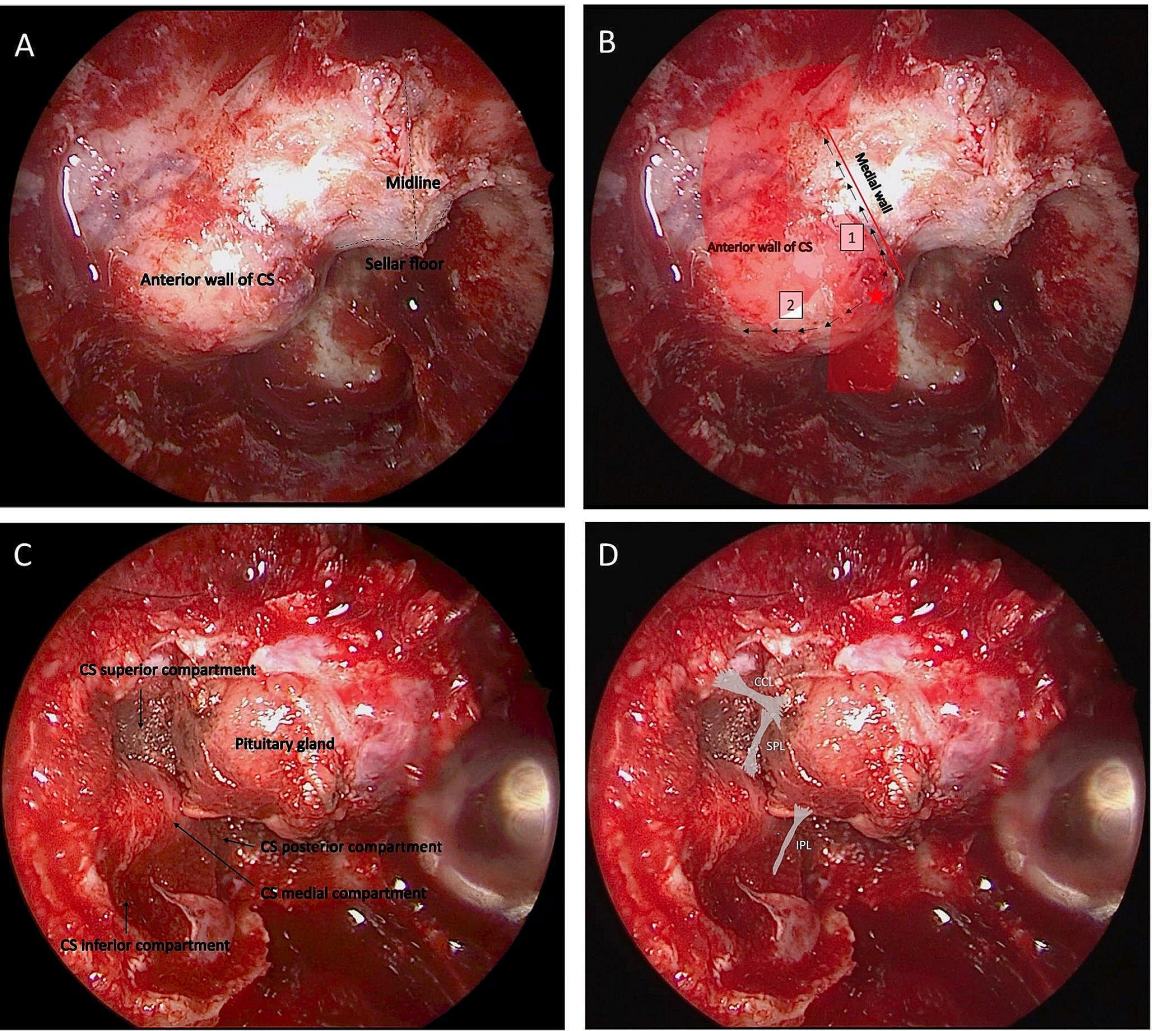
(A) Intraoperative view showing the wide exposure of the anterior wall of the cavernous sinus that is prominent due to the lesion. (B) Schematic representation of the sequence of dural cuts for opening the anterior wall of the cavernous sinus. The anterior wall is opened at the lowest and medial aspect of the anterior wall where the carotid bends posteriorly to form the posterior genu. It is, subsequently, largely opened in a superior direction parallel to the medial wall of the CS and lateral direction. (C) intraoperative view showing the compartments of the CS after complete tumor resection. (D) Schematic representation of the inferior (IPL) and superior (SPL) parasellar ligaments and the carotid-clinoid ligament (CCL)

To access the anterior wall of the CS a wide sphenoidotomy is performed to allow for the necessary lateral exposure(Fig. 2A).

Septations are removed down to the sella or over the ICA avoiding any excessive torquing of the bone fragments. The sellar bone is then removed from CS to CS and from the sellar floor inferiorly to the tuberculum sella superiorly with a high-speed diamond bit drill and a Kerrison rongeur. On the side where the tumor extends into the CS, the bone is carefully removed from the anterior wall of the CS. A carotid Doppler is used to methodically map out the ICA in relation to the anterior CS wall. After doppler verification of ICA position, the anterior wall of the CS is opened using a right-angled feather blade at the lowest and medial aspect of the anterior wall where the ICA bends posteriorly to form the posterior genu. Low-flow venous bleeding can be easily controlled with light packing with Floseal.

**Fig. 3:**
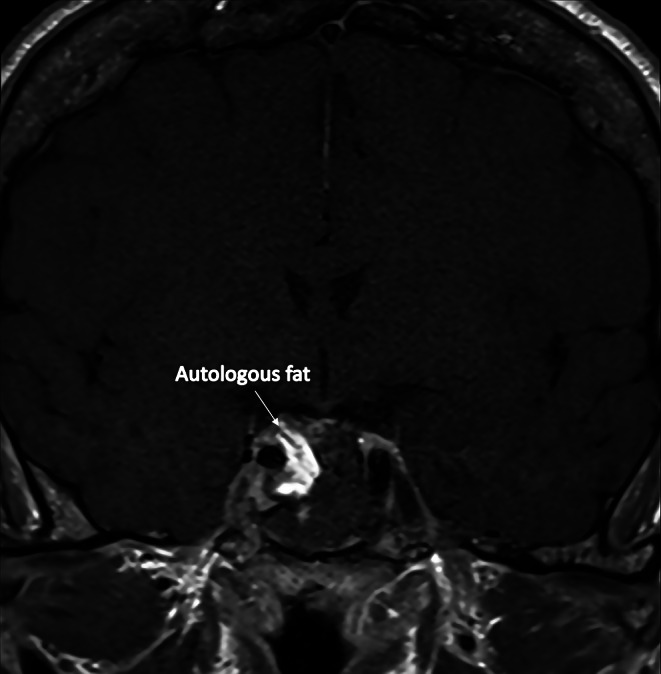
Post-operative T1W coronal MR image showing the placement of autologous fat in the resection lodge covering the carotid artery

Under direct visual control of the ICA, the anterior CS wall is largely opened in a lateral direction and superior direction parallel to the medial wall of the CS to provide adequate visualization of the CS contents and remove tumor off the inferior compartment (Fig. 2B and C). The IPL is frequently visualized at this level and easily dissected and cut. Once the tumor is visualized, a systematic dissection is performed to define its limits and separe it from the ICA and medial wall of the CS. The tumor in the medial and inferior compartment is removed progressively. During dissection the PPL and SPL are visualized, dissected, coagulated and sectioned so as the tumor can be mobilized from the medial surface of the cavernous ICA opening up the superior and posterior compartment of the CS (Fig. 2D) [10]. Tumor can be dissected off these two compartments paying attention to the inferior hypophyseal artery, which usually courses across the base of the posterior clinoid. This last, if necessary, can be coagulated and sharply cut to avoid avulsion. The final ligamentous attachments tethering the medial wall and the tumor to the roof of the CS is CCL, which is cut after being coagulated using sharp dissection. Final hemostasis is achieved, autologous fat, previously harvested at the abdominal level, is placed at the level of the resection lodge so as to avoid exposure of the ICA (Fig. 3).

## Indication

Surgery is the main treatment for most functional pituitary adenoma with oncologic control in 60–90% of cases depending on published series [8]. Invasion of the CS is one of the limiting factors for total resection for PitNet tumors with cure rates less than 30% [8]. Extended approaches may be considered in selective and well-studied cases of secreting adenomas and only in tertiary centers.

## Limitations

In the case of extension to the level of the lateral compartment of the CS or with invasion of its roof which prevents a total resection [9], the benefit of partial resection of the intracavernous component must be rationally weighed. The presence of interventional radiology are mandatory.

## How to avoid complications

- A detailed knowledge of CS anatomy and familiarity with this surgical approach acquired in the laboratory is essential.
- The anatomical location of the sixth cranial nerve within the CS and its relationship to the ICA should always be kept in mind during tumor resection.
- Meticulous preoperative study of the radiologic anatomy of the tumor and its relationship to the CS and ICA is essential.
- Proper planning of surgical access (contralateral vs. transpterygoid) is critical to access the anterior wall of the CS without anatomic restrictions and in a direct manner.
- Proper instrumentation is critical to decrease the risks of vascular injury. This includes a surgical navigation system, a scalpel with blunt-tip blade to dissect the anatomic plane between the medial wall of the CS and the ICA and excise the parasellar ligaments.
- The combined use of excellent anatomic knowledge, intraoperative Doppler, and neuronavigation together represent the best strategy to minimize the risk of complications.
- The use of microdoppler is essential to locate the exact position of the cavernous ICA.
- it is important to obtain adequate venous control for proper visualization.

## Specific information to give to the patient about surgery and potential risks

Resection of the intracavernous tumor and the medial wall of the CS is associated with disease remission in nearly 95% of cases [4, 7] of functioning PitNet. However, this technique has an increased risk of nerve injury (4.8% of cases) and potential risk of carotid injury [2], which is why the risk-benefit balance must be rigorously evaluated and discussed with the patient. The preoperative and postoperative management of patients operated on for a pituitary lesion was detailed recently in one of our papers [3].

### 10 key points summary

1. Extended approaches may be considered in selective and well-studied cases of secreting adenomas and only in tertiary centers.
2. A detailed knowledge of CS anatomy and familiarity with this surgical approach acquired in the laboratory is essential.
3. For tumors projecting predominantly to one side of the sella and extending into the CS, a proper planning of surgical access (contralateral vs. transpterygoid) is critical to access the anterior wall of the CS without anatomic restrictions and in a direct manner.
4. To access the anterior wall of the CS a wide sphenoidotomy is performed and the sellar bone is removed from CS to CS and from the sellar floor inferiorly to the tuberculum sella superiorly to allow for the necessary lateral exposure without restrictions.
5. Proper instrumentation is critical to decrease the risks of vascular injury. This includes a surgical navigation system, a carotid Doppler and a scalpel with blunt-tip blade to dissect the anatomic plane between the medial wall of the CS and the ICA and excise the parasellar ligaments.
6. A carotid Doppler is critical for methodically mapping out the ICA in relation to the anterior CS wall.
7. The anterior wall of the CS is opened using a right-angled feather blade at the lowest and medial aspect of the anterior wall where the ICA bends posteriorly to form the posterior genu.
8. The inferior hypophyseal artery must be identified during dissection so as to avoid its avulsion during tumor resection.
9. The presence of an interventional neuroradiologist is mandatory given the potential risk of vascular injury.
10. Autologous fat or pediculated flap is placed at the level of the resection lodge so as to avoid exposure of the ICA.

## Electronic supplementary material

Below is the link to the electronic supplementary material.

Supplementary Material 1

## References

[1] Berhouma M, Messerer M, Jouanneau E (2012) Occam’s razor in minimally invasive pituitary surgery: tailoring the endoscopic endonasal uninostril trans-sphenoidal approach to sella turcica. Acta Neurochir (Wien) 154:2257–2265. 10.1007/s00701-012-1510-2 23053285 10.1007/s00701-012-1510-2

[2] Cossu G, Atkins T, Hajdu SD, Puccinelli F, Daniel RT, Messerer M (2020) Hemorrhagic direct traumatic carotid-cavernous fistula during endoscopic transsphenoidal surgery: intraoperative management and endovascular treatment. Neurosurg Focus Video 2:V1. 10.3171/2020.4.FocusVid.19918 36284789 10.3171/2020.4.FocusVid.19918 PMC9542588

[3] Cossu G, Belouaer A, Kloeckner J, Caliman C, Agri F, Daniel RT, Gaudet JG, Papadakis GE, Messerer M (2023) The enhanced recovery after surgery protocol for the perioperative management of pituitary neuroendocrine tumors/pituitary adenomas. Neurosurg Focus 55:E9. 10.3171/2023.9.FOCUS23529 38039521 10.3171/2023.9.FOCUS23529

[4] de Macedo Filho LJM, Diogenes AVG, Barreto EG, Pahwa B, Samson SL, Chaichana K, Quinones-Hinojosa A, Almeida JP (2022) Endoscopic endonasal resection of the Medial Wall of the cavernous sinus and its impact on outcomes of pituitary surgery: a systematic review and Meta-analysis. Brain Sci 12. 10.3390/brainsci12101354 10.3390/brainsci12101354 PMC9599381 36291288

[5] Fernandez-Miranda JC, Zwagerman NT, Abhinav K, Lieber S, Wang EW, Snyderman CH, Gardner PA (2018) Cavernous sinus compartments from the endoscopic endonasal approach: anatomical considerations and surgical relevance to adenoma surgery. J Neurosurg 129:430–441. 10.3171/2017.2.JNS162214 28862552 10.3171/2017.2.JNS162214

[6] Messerer M, Cossu G, Daniel RT (2019) Endoscopic approach for a delayed post-traumatic ethmoidal mucocele: a technical note. Br J Neurosurg 33:107–109. 10.1080/02688697.2017.1297770 28293965 10.1080/02688697.2017.1297770

[7] Mohyeldin A, Katznelson LJ, Hoffman AR, Asmaro K, Ahmadian SS, Eltobgy MM, Nayak JV, Patel ZM, Hwang PH, Fernandez-Miranda JC (2022) Prospective intraoperative and histologic evaluation of cavernous sinus medial wall invasion by pituitary adenomas and its implications for acromegaly remission outcomes. Sci Rep 12:9919. 10.1038/s41598-022-12980-1 35705579 10.1038/s41598-022-12980-1 PMC9200976

[8] Starnoni D, Daniel RT, Marino L, Pitteloud N, Levivier M, Messerer M (2016) Surgical treatment of acromegaly according to the 2010 remission criteria: systematic review and meta-analysis. Acta Neurochir (Wien) 158:2109–2121. 10.1007/s00701-016-2903-4 27586125 10.1007/s00701-016-2903-4

[9] Starnoni D, Cossu G, Messerer M, Daniel RT (2022) Transcavernous Approach to the Anteromedial Triangle for residual functional pituitary adenoma. J Neurol Surg B Skull Base 83:e630–e631. 10.1055/s-0041-1727119 36068893 10.1055/s-0041-1727119 PMC9440874

[10] Truong HQ, Lieber S, Najera E, Alves-Belo JT, Gardner PA, Fernandez-Miranda JC (2018) The medial wall of the cavernous sinus. Part 1: Surgical anatomy, ligaments, and surgical technique for its mobilization and/or resection. J Neurosurg 131:122–130. 10.3171/2018.3.JNS18596 30192192 10.3171/2018.3.JNS18596

